# Cytotoxic Chemotherapy as an Immune Stimulus: A Molecular Perspective on Turning Up the Immunological Heat on Cancer

**DOI:** 10.3389/fimmu.2019.01654

**Published:** 2019-07-17

**Authors:** James W. Opzoomer, Dominika Sosnowska, Joanne E. Anstee, James F. Spicer, James N. Arnold

**Affiliations:** Faculty of Life Sciences and Medicine, School of Cancer and Pharmaceutical Sciences, King's College London, Guy's Hospital, London, United Kingdom

**Keywords:** tumor, chemotherapy, immunotherapy, chemo-immunotherapy, microenvironment

## Abstract

Cytotoxic chemotherapeutics (CCTs) are widely used in the treatment of cancer. Although their mechanisms of action have been best understood in terms of targeting the apparatus of mitosis, an ability to stimulate anti-tumor immune responses is increasing the recognition of these agents as immunotherapies. Immune checkpoint blockade antibodies neutralize important, but specific, immune-regulatory interactions such as PD-1/PD-L1 and CTLA-4 to improve the anti-tumor immune response. However, CCTs can provide a broad-acting immune-stimulus against cancer, promoting both T-cell priming and recruitment to the tumor, which compliments the effects of immune checkpoint blockade. A key pathway in this process is “immunogenic cell death” (ICD) which occurs as a result of tumor cell endoplasmic reticulum stress and apoptosis elicited by CCTs. ICD involves a series of non-redundant signaling events which break tolerance and license anti-tumor antigen-specific T-cells, allowing CCTs to act as “*in situ*” tumor vaccination tools. Not all responses are tumor cell-intrinsic, as CCTs can also modulate the broader tumor microenvironment. This modulation occurs through preferential depletion of stromal cells which suppress and neutralize robust anti-tumor immune responses, such as myeloid cell populations and Tregs, while effector CD8^+^ and CD4^+^ T-cells and NK cells are relatively spared. The immune-stimulating effects of CCTs are dependent on chemotherapy class, dose and tumor cell sensitivity to the agent, highlighting the need to understand the underlying biology of these responses. This mini review considers the immune-stimulating effects of CCTs from a molecular perspective, specifically highlighting considerations for their utilization in the context of combinations with immunotherapy.

## Introduction

**Figure 1:**
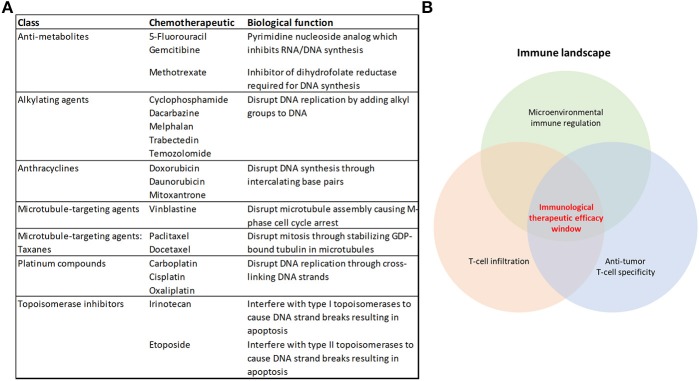
Cytotoxic chemotherapeutics and the therapeutic response to immunotherapy. **(A)** Table summarizing the chemotherapeutics discussed in this review, their broader class and biological function **(B)** Some of the overarching requirements needed for an effective anti-tumor immune response that will achieve immunological tumor control.

Chemotherapy has been utilized for the treatment of cancer for over 70 years (1), and cytotoxic chemotherapeutics (CCTs) form part of the treatment regimen for many patients with cancer. However, as single agents or as chemotherapy combinations, they rarely represent cures for advanced-stage disease (2), and the efficacy requires improvement in adjuvant and radiotherapy-combination settings. These classes of drugs target replicating malignant cells by disrupting features core to the cell cycle, including DNA replication and the inhibition of the dynamic processes of mitosis (Figure 1A). Despite their use in the treatment of cancer for their cytotoxic properties, there is mounting evidence that they also promote an anti-tumor immune response (3–10). Given these observations, there may be significant opportunities for the inclusion of CCTs in immunotherapy regimens.

Immunotherapy has represented a paradigm shift in the way that oncologists approach the treatment of cancer. Unprecedented clinical benefit has been achieved in a variety of cancers using Immune checkpoint inhibitors (ICIs) that targets immune regulation of T-cell responses, such as antibodies which neutralize PD-1 or its ligand PD-L1, or CTLA-4 (11). However, there remains a majority of patients (up to 60–70%) who are refractory to the current therapies or acquire resistance (12, 13), and there is a notable variation in patient responses to ICIs across different tumor types (14, 15). Combining immune checkpoint blockade therapies improves patient outcomes using the currently-available therapies (12, 16, 17). Also, the number and location of anti-tumor cytolytic T-lymphocytes (CTLs) in the tumor microenvironment dictate the response to these therapies (18–23) (Figure 1B). Harnessing the broad immune-stimulating capabilities of CCTs in combination with immune checkpoint therapies has shown great promise (17, 24), with improved clinical outcomes (25–29). There are also further opportunities for CCTs to be used more broadly in combination with the growing range of immunotherapies (30).

This mini review considers our current mechanistic understanding of the immune-stimulating capabilities of CCTs for improving the anti-tumor immune response and proposes that these drugs should be considered a versatile therapeutic option in the immunotherapy repertoire.

## Immune Priming: Immunogenic Death of Tumor Cells

The observation that cell death, in the absence of infection, can result in CD8^+^ T-cell responses against “dead cell” antigens, has been an area of significant research interest and has been coined “immunogenic cell death” (ICD) (6, 31). Key steps leading to, and dictating whether, an anti-tumor immune response occurs have been identified in an elegant series of studies by the field. CCTs have been demonstrated to be at least one initiator/potentiator of this process, which is not seen with all chemotherapy drugs, but has been most commonly characterized using anthracyclines (32), platinum compounds (19), and alkylating agents (33).

ICD requires an induction of endoplasmic reticulum (ER) stress and autophagy in the tumor cell by the CCT (34–36). Through the ER stress response, the reticular chaperone calreticulin (CRT) is presented on the cell surface as part of a complex with the disulphide isomerase ERp57, as an early pre-apoptotic event, preceding even the presentation of apoptotic markers such as phosphatidylserine (34, 37). Phagocytic cells then detect surface-presented CRT/ERp57 using CD91 (LDL-receptor related protein/α2-macroglobulin receptor) which provides a potent “eat me signal” for phagocytic engulfment of the cell (38, 39), which is pivotal for the generation of a subsequent immune response (40, 41). Interestingly, DNA damage by anthracyclines is not the initiating signal for this response, as enucleated cells (cytoplasts) exposed to mitoxantrone present surface CRT and are phagocytosed by dendritic cells (DCs) at an equivalent rate to that of nucleated cells (39). Cisplatin has also been demonstrated to be capable of inducing a DNA damage-independent ER-stress response in enucleated cells through a pathway which required calcium and the calcium-dependent protease calpain (42).

During the blebbing phase of apoptosis, release of adenosine triphosphate (ATP) from the dying cell potentiates ICD and provides a “find me” signal which attracts DCs and macrophages to the site (43), and stimulates their maturation (44). ATP signaling through the purinergic receptor P2X_7_ on phagocytic cells triggers activation of the NOD-like receptor family, pyrin domain containing-3 protein (NLRP3)-dependent caspase-1 activation complex (inflammasome), which subsequently results in the release of the pro-inflammatory cytokine IL-1β (32, 45). IL-1β then attracts IFN-γ secreting CTLs to the tumor site via IL-17-producing γδ T-cells (41). Interestingly, there is evidence that the IFN-γ expression by the CD8^+^ tumor infiltrating lymphocytes (TILs) is vital to the anti-tumor response elicited through ICD, as the immunological control of tumor growth by oxaliplatin has been demonstrated to be independent of perforin, and IFN-γ-dependent (32).

In the latter stages of cell death, there is a release of damage-associated molecular patterns (DAMPs) (46), which license ICD. Two key ICD-related DAMPs have been identified as nuclear non-histone chromatin protein high mobility group box 1 (HMGB1) (47) and surface heat shock protein 90 (HSP90) (48), which are capable of signaling as endogenous ligands of Toll-like receptor-4 (TLR-4) on DCs, leading to their processing and presentation of tumor-associated antigens, rendering the cell death immunogenic rather than tolerogenic. As clinical support for these observations, loss-of-function alleles of the *TLR4* gene are a negative predictor of benefit from adjuvant chemotherapy with anthracyclines or oxaliplatin (47). HMGB1 has also been demonstrated to facilitate the recruitment of neutrophils and natural killer (NK) cells into the tumor microenvironment of a xenograft model of breast cancer in athymic mice, where both populations were required for cyclophosphamide to control tumor growth (49). ICD requires multiple non-redundant licensing steps, as either blockade of surface CRT exposure (39, 40), HMGB1-dependent TLR4 signaling (47), or autophagy-depended ATP release (35) severely compromises ICD. Since robust immune-mediated tumor killing can be unmasked in a subset of patients through the use of ICIs in the absence of CCTs (50), and the rare cases of spontaneous tumor remission (51), it is likely that ICD can also occur without the need for an adjuvant. This is also supported in preclinical models where spontaneous anti-tumor immune responses, in the absence of therapeutic interventions, occur (52–54). However, harnessing CCTs as well as some targeted agents (6), to elicit ICD provide powerful therapeutic tools for use in patients with undetectable or weak anti-tumor immune responses (31). Further to this, tumors carrying a high mutational burden provide the immune system with neoantigens to mount such responses against, underscored by the observation that tumor mutational burden correlates with clinical benefit of ICIs targeting PD-1 and CTLA-4 (55–57). However, even non-mutated proteins can be antigenic when inappropriately expressed, such as the cancer/testis antigens (58). Pharmacodynamic markers which monitor for evidence of an ICD-type response to CCTs might provide useful information in tailoring treatment regimens to improve ICD as cancer therapy becomes more personalized (Figure 1B).

## Trafficking and Infiltration of T-Cells

There is a clear link between the presence of TILs and progression free and overall survival in a variety of cancers (59–64). The prevalence of TILs, the “Immunoscore,” has been shown to reliably predict the risk of recurrence (3 year risk of recurrence, high vs. low Immunoscore hazard ratio 0.2, 95% confidence interval 0.1–0.38, *p* < 0.0001) in a large international cohort study of patients with TNM stage I-III colon cancer (65). The presence of TILs also correlates with the clinical response to ICIs targeting both PD-1 (66) and CTLA-4 (67) receptors. Tumor microenvironments which lack T-cell infiltration have become known as “immunologically cold,” which can result due to either exclusion mechanisms or a lack of a TIL-type of inflammation to attract these cells to the site (18, 68, 69). However, a potentially important biological response to CCTs is their ability to initiate a T-cell influx into the tumor microenvironment (10, 70–74) (Figure 1B). A high CD8/Foxp3 TIL ratio post-neoadjuvant chemotherapy is predictive for improved relapse free and overall survival in patients with breast cancer (75). Paclitaxel, at a dose of 200 mg/m^2^ every 2 weeks for 4 cycles, was shown to improve TIL numbers in 7 out of 21 patients with breast cancer (70). Intriguingly, in this study, T-cell infiltration tended to occur in patients whose tumors had a strong apoptotic response acutely (96 h) after receiving the first dose of paclitaxel (70). Others have observed chemotherapy-dependent CD8^+^/CD4^+^ T-cell infiltration in response to paclitaxel and gemcitabine in a murine model of ovarian cancer (74) and 5-FU in a murine model of breast cancer (10). In models of melanoma, temozolomide improved TIL recruitment into the tumor in a CXCR3-dependent manner (72). Others have also shown CXCR3 to be non-redundant to T-cell recruitment into the tumor (76). *In vitro* exposure of melanoma cell lines to either temozolomide, cisplatin, or dacarbazine resulted in their expression of T-cell chemokines CCL5, CXCL9, and 10. However, the response was not predictable, as different CCTs promoted T-cell chemokine expression in different cell lines (72), suggesting that tumor cell sensitivity is an important variable in this process. In another study, doxorubicin was shown to induce a rapid TLR3-dependent expression of interferon β1 (IFN-β1) from tumor cells, which was triggered by the release of self RNA by chemotherapy-stressed or dying cells (77). IFN-β1 then signaled in both a paracrine and autocrine fashion to promote the IFN-α/β receptor-dependent release of the T-cell chemokine CXCL10, alongside a concurrent expression of MHCI (77). The CCT-induced expression of tumor cell MHCI has also been shown by others using ovarian cancer cell lines exposed to gemcitabine, paclitaxel or carboplatin (74), and renders the tumor cells more susceptible to CTL killing (78). Using CCTs to promote T-cell infiltration into the tumor and convert previous immunological “cold” microenvironments “hot,” is an important attribute of these drugs. However, as the response appears to be both dependent on the tumor cell and the chemotherapy it is exposed to (72), how to anticipate the response for efficient pairing of chemotherapy to each patient has yet to be established.

## The Response of the Stroma

The tumor stroma, the heterogenous population of non-cancerous cells, some of which can facilitate tumor progression, play significant roles both directly and indirectly in modulating the response to chemotherapeutics. Examples of populations that have been demonstrated to suppress the infiltration or activity of anti-tumor CD8^+^ T-cells include cancer associated fibroblasts (18, 54), tumor associated macrophages (TAMs) (10), myeloid-derived suppressor cells (MDSCs) (79, 80), and Tregs (81). There is evidence that CCTs can selectively target immune suppressive cell populations, for example preferential depletion of Tregs in response to paclitaxel (82), cyclophosphamide (83), or temozolomide (84) treatment. MDSCs have been shown to be preferentially depleted by doxorubicin (85, 86) and 5-FU (85), and TAMs by gemcitabine (9). CCTs can also activate NK cells (87), but also concurrently render tumor cells more susceptible to NK cell-mediated lysis, such as through promoting the expression of B7-H6, the ligand for the NK cell activating receptor NKp30, on the tumor cell surface (88). CCTs can also modulate MDSC differentiation/polarization, where paclitaxel promoted monocytic MDSC differentiation into a more DC-like phenotype (89) and docetaxel promoted their differentiation into a more pro-inflammatory macrophage phenotype (90).

TAMs are a prevalent cell type in the tumor and have been demonstrated to modulate the therapeutic efficacy of CCTs (10, 37, 91). Many of these effects are due to their immune suppressive capabilities (10). In the spontaneous MMTV-PyMT murine model of breast cancer, macrophage expression of heme oxygenase-1 (HO-1), an enzyme responsible for the breakdown of heme to generate the biologically active products biliverdin, ferrous iron (Fe^2+^) and carbon monoxide (CO) (92), was demonstrated to play a pivotal role in suppressing an anti-tumor immune response generated by 5-FU (10). However, TAM secretion of IL-10 has also been demonstrated to be important in suppressing paclitaxel-elicited CD8^+^ T cell responses indirectly by suppressing IL-12 release from DCs in the tumor microenvironment (91). Macrophage can also promote tumor cell survival in response to CCTs through their secretion of cathepsins B and S (93). Furthermore, tumor-polarized TAMs, in murine models of pancreatic ductal adenocarcinoma, have been demonstrated to release deoxycytidine which can be taken up by tumors cells to directly compete with gemcitabine, hindering the drug's efficacy (94). TAMs are highly plastic in their phenotype and biological response, and paclitaxel can skew the polarization to a pro-inflammatory phenotype through activation of TLR4 via an interaction of paclitaxel with the extracellular accessory protein MD2 (95, 96). However, this response is a murine-specific phenomenon, as paclitaxel binds mouse but not human MD2 (95). Nevertheless, another member of the taxane family, docetaxel, was shown to influence human macrophage polarization toward a more pro-inflammatory state characterized by increased HLA-DR, CD86 expression and their secretion of IL-1β and IL-8 (97).

Peripheral macrophages in the spleen also play a role in suppressing the apoptotic response of tumor cells distal to the spleen. Intravenous infusion of bone marrow-derived mesenchymal stromal cells exposed to carboplatin, oxaliplatin, and cisplatin released platinum-induced fatty acids (PIFAs), and conferred tumor resistance to platinum-based chemotherapeutics in murine models (98). Splenic macrophages (F4/80^+^ CD11b^low^) which had become activated by PIFAs via leukotriene B4 receptor 2 (BLT2) secreted polyunsaturated lysophosphatidylcholines (LPCs) which were capable of altering the DNA damage response in the distant tumor, and conferred therapy resistance (99).

The release of IL-1β by DCs in response to doxorubicin treatment plays an important role in recruiting IL-17 producing γδ T-cells which subsequently recruit anti-tumoral IFN-γ expressing αβ CD8^+^ T-cells into the tumor microenvironment (41). Conversely, expression of IL-1β by MDSCs in response to either gemcitabine or 5-FU was demonstrated to induce IL-17 expression by CD4^+^ T cells, which suppressed the chemotherapy-dependent control of tumor growth (100). Interestingly, IL-17 from γδ T-cells can induce both the suppressive activity of MDSCs and the tumor-derived release of CXCL5, which recruits MDSCs (101). As such, when MDSCs are present, and activated by IL-17, potentially their immune suppressive effects override the pro-inflammatory anti-tumor response of IL-17. These mechanisms provide examples of the importance of the immune landscape of the tumor when considering chemotherapy-elicited immune responses.

## The Importance of Dose and Schedule

CCTs target all replicating cells, leading to predictable effects on normal tissues with proliferating cell populations. For example, cytopenias commonly result from the impact of CCTs on the bone marrow, where pools of replicating cells drive hematopoiesis. One study in mice, analyzed the changes in gene expression after administration of cyclophosphamide and found bone marrow, spleen, and blood PBMCs, had 1123, 868, and 1083 differentially regulated genes respectively 1–2 days post administration, which in the bone marrow and PBMC fraction returned back to baseline at day 5 post-administration (102). In the clinic, CCTs are largely administered at the maximum tolerated dose which can be immunosuppressive as a result of myelosuppression. However, the recovery phase from chemotherapy-elicited lymphopenia can be an important window where anti-tumor immune responses become potentiated (103). Furthermore, low dose, but dose dense (“metronomic”), administration of paclitaxel and cisplatin in a subcutaneous HM-1 ovarian cancer model resulted in CD8^+^ T-cell dependent control of tumor growth that was superior to that observed at the maximum tolerated dose (104). The mechanistic understanding of why low dose metronomic chemotherapy regimens are generally more immune-stimulating is not entirely clear. However, maximum tolerated dose regimens are associated with a loss of CD8^+^ and CD4^+^ T-cells and NK cells from the tumor microenvironment, and pro-tumoral activation of cancer-associated fibroblasts (105). Whereas, low dose regimens preferentially target MDSCs (CD11b^+^ Gr-1^+^) and Tregs while concurrently increasing IFN-γ expressing T-cells (104, 106, 107), and activating NK cells (108). In a phase III trial of patients with advanced ovarian cancer a low dose metronomic paclitaxel regimen alongside carboplatin resulted in a significantly lengthened survival of 28 months compared to 17.2 months on the standard treatment regimen (hazard ratio 0.71; 95% confidence interval 0.58–0.88, *p* = 0.0015) (109). This observation in ovarian cancer patients has also been supported by others (110), however, the beneficial effects of the low dose dose-dense treatment regimen was only seen in those patients that had not received bevacizumab (110). Others have suggested medium intermittent dose regimens to strike the optimal balance between the cytotoxic roles of these drugs and the immune-stimulating effects (111). There is evidence to suggest that the dose and schedule are both important variables to efficiently harness the immune-stimulating effects of CCTs. However, further preclinical studies focusing on the biological mechanisms which account for dose and schedule effects are needed. It is likely that the optimal CCT dose is not equivalent for all patients due to tumor cell characteristics, efficiency of drug delivery, and microenvironment heterogeneity. Understanding how to clinically evaluate, and potentially predict, the optimal CCT regimen is an important question to be addressed.

## Conclusions

**Figure 2:**
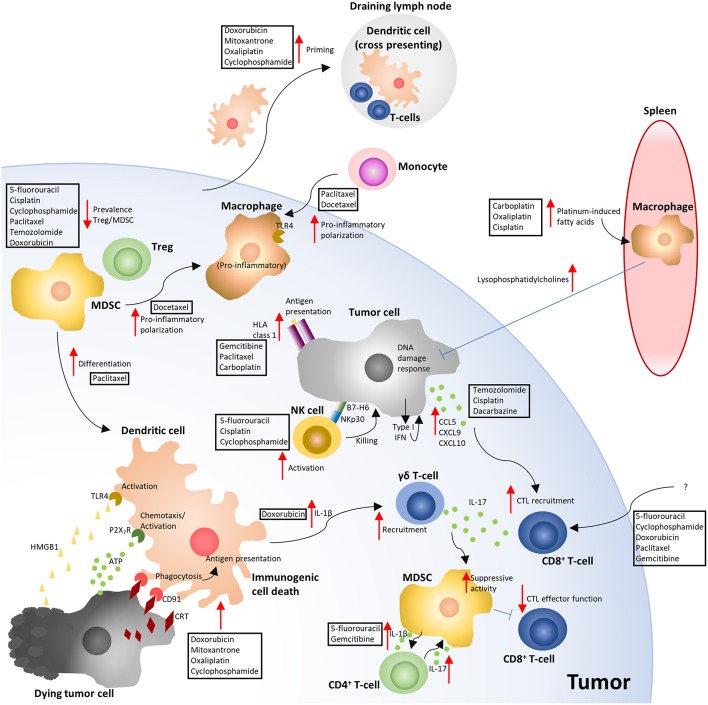
Overview of the immune-modulating effects of cytotoxic chemotherapy. The depicted mechanisms, and chemotherapies shown to elicit these responses, summarize the results of the studies highlighted in the manuscript text. Red arrows indicate either an increased (pointing up) or decreased (pointing down) response. Blue flat ended lines represent an inhibitory effect relating to the mechanism depicted. The text boxes positioned near the arrows indicate the CCTs that were described to elicit the response. ATP, Adenosine triphosphate; CRT, Calreticulin; CTL, Cytotoxic T-lymphocyte; HLA, Human leukocyte antigen; HMGB1, High mobility group box 1; MDSC, Myeloid derived suppressor cell; NK, Natural killer; TLR4, Toll like receptor 4; Treg, T-regulatory cell (6, 10, 32, 33, 36, 40, 41, 47, 49, 70–74, 76, 78, 83, 86, 88–90, 96–98, 100, 101, 104, 105).

CCTs have an intriguingly broad ability to modulate the anti-tumor immune response, providing benefit via several distinct mechanisms (Figure 2) that influence the response to immunotherapy (Figure 1B). Clinical outcomes with either CCTs or immunotherapies alone leave significant room for improvement, so there is a good rationale for exploring possible synergistic effects on the tumor microenvironment of the two combined modalities. The clinical evidence for combining ICIs with chemotherapy is already robust and supported by randomized trials (112, 113), comprehensively reviewed by (114). However, as the biological response of a tumor cell varies according to the CCT that it is exposed to (9, 72, 78), understanding how to predict these responses becomes increasingly important. CCT-elicited cell stress and apoptosis is clearly linked to ICD (6), and non-lethal “stress” can also render tumor cells vulnerable to T-cell killing (115). How to balance CCT dose to efficiently elicit the required immune-stimulating effects (6, 115, 116), preferentially eliminate immune suppressive cells (82–86), and avoid lymphodepletion (104, 106), highlight some of the potential variables in moving to an efficient use of these drugs as immunotherapies in a more personalized manner. The emerging importance of gut microbiota in dictating the efficacy of both ICIs (117, 118) and chemotherapy (119, 120) provide further confounding factors for rationally predicting clinical benefit. However, as our knowledge of the biological mechanisms underlying the immune-stimulating properties of CCTs continues to deepen, their utilization as immunotherapies with broad immune-stimulating effects offer significant promise for improving the number of patients benefiting from immunotherapy.

## Author Contributions

JO, DS, JEA, JS, and JNA wrote the manuscript.

### Conflict of Interest Statement

The authors declare that the research was conducted in the absence of any commercial or financial relationships that could be construed as a potential conflict of interest.
